# microRNAs in soft tissue sarcoma: State of the art and barriers to translation

**DOI:** 10.18632/oncotarget.28754

**Published:** 2025-07-16

**Authors:** Elizaveta K. Titerina, Alessandro La Ferlita, Joal D. Beane

**Keywords:** soft tissue sarcoma, liposarcoma, microRNA, small non-coding RNA, cancer biomarkers

Soft-tissue sarcomas (STS) represent a diverse group of more than 50 rare cancer subtypes that originate from mesenchymal tissues. This diversity has led to considerable challenges in diagnosing and treating individual subtypes, necessitating a personalized approach for each tumor type based on its specific molecular mechanisms. Our recent review explores the molecular processes by which small non-coding RNAs (sncRNAs), particularly the well-studied microRNAs (miRNAs), contribute to oncogenic or tumor-suppressive effects in sarcoma development. We highlight how these molecules influence cell proliferation, apoptosis, and metastasis in the formation and progression of STS. We did this while also considering their potential as biomarkers for early detection and as promising targets for therapy. However, significant hurdles remain in the implementation of miRNA-based therapies.

Beyond the role of miRNAs in modulating key cancer pathways, their efficacy as therapeutic agents remain uncertain; however, they are emerging as promising biomarkers for patients with STS. Specifically, miRNAs have been shown to correlate with patients’ response to systemic therapy. For example, miR-17-92 and miR-106b-25 clusters have been associated with sensitivity or resistance to eribulin in STS [1]. Additionally, the expression of miR-761 serves as a biomarker for resistance to pazopanib in synovial sarcoma [2]. Since miRNAs are relatively stable in biological fluids, such as blood and saliva, they represent an alternative for minimally invasive and non-invasive clinical biomarkers [3]. Recent studies advocate for standardized protocols in blood collection, sample processing, and storage to preserve miRNA integrity, thereby enhancing their utility in diagnosing and monitoring diseases [3–5].

Expanding on the established role of miRNAs as biomarkers, their specific application in liposarcoma (LPS) provides insights into distinguishing between sarcoma types that are often challenging to differentiate histologically and genetically. This is particularly critical for diagnosing benign lipomas and adipocytic sarcomas [6]. LPS represents a heterogeneous group of malignancies with distinct clinical, histological, and molecular features, making them challenging to diagnose. By implementing specific miRNA expression patterns into diagnostic arsenal, clinicians could achieve more accurate diagnoses for guiding appropriate treatment strategies. For instance, the presence of miR-25-3p and miR-92a-3p in extracellular vesicles (EV) from serum has been shown to effectively differentiate well-differentiated (wdLPS) and dedifferentiated LPS (ddLPS) from healthy controls [7]. Additionally, miRNAs such as miR-1246, miR-4532, miR-4454, miR-619-5p, and miR-6126 are highly expressed in the serum and tissues of patients with ddLPS, highlighting their potential as significant biomarkers [8]. Overall, miRNAs could serve as a non-invasive diagnostic tool to serve in detecting and classifying liposarcoma subtypes.

Despite their potential as biomarkers, significant challenges hinder the clinical application of miRNAs in treating STS. No miRNA-based therapies have been trialed in STS patients for diagnosis or treatment, primarily due to their dual role in gene regulation—miRNAs can function as tumor suppressors and promoters depending on the cellular context. This complexity makes identifying effective targets a continuing area of research. Additionally, practical challenges with miRNA delivery or their inhibitors have hampered translational efforts. While viral vectors such as lentiviruses and adeno-associated viruses are efficient, they pose risks of immunogenicity and cytotoxicity [9]. Consequently, non-viral methods such as lipid-based nanocarriers (LNPs), polymeric nanoparticles, and extracellular vesicles (EVs) are being explored for their safety and targeted delivery capabilities [10]. These systems are particularly promising, with LNPs leading by offering customizable solutions to minimize off-target effects. For instance, LNPs can be engineered to target specific organs, and EVs can be designed to home in on cancer cells by recognizing specific markers. These and other novel approaches are being utilized to refine miRNA delivery techniques to enhance therapeutic efficacy, balance delivery effectiveness with minimal side effects, and broaden the scope of miRNA applications in clinical settings [11].

In summary, miRNAs are pivotal in regulating gene expression in STS and have been recognized as crucial biomarkers for the diagnosis, prognosis, and therapeutic decision-making in patients. Despite the existing challenges in miRNA-based therapy, such as complex gene regulation roles and delivery issues, these molecules have substantial potential to transform the treatment of STS. If these challenges are addressed, miRNA-based strategies could become an integral part of clinical practice in patients with STS.
